# Granulocytic anaplasmosis in captive ring-tailed lemur (*Lemur catta*) in Poland

**DOI:** 10.1186/s12917-021-02827-8

**Published:** 2021-03-12

**Authors:** Łukasz Adaszek, Anna Wilczyńska, Jerzy Ziętek, Marcin Kalinowski, Oliwier Teodorowski, Dagmara Winiarczyk, Maciej Skrzypczak, Stanisław Winiarczyk

**Affiliations:** 1 Department of Epizootiology and Infectious Diseases, Faculty of Veterinary Medicine, University of Life Sciences in Lublin, 30 Głęboka St. 20-612, Lublin, Poland; 2 Veterinary Clinic “Teodorowscy” in Mikołów, Mikołów, Poland; 3 Department and Clinic of Animal Internal Diseases, Faculty of Veterinary Medicine, University of Life Sciences, 30 Głęboka St. 20-612, Lublin, Poland; 4 Second Department of Gynecology, Prof. F. Skubiszewski University School of Medicine, Lublin, Poland

**Keywords:** *Anaplasma phagocytophilum*, Ring-tailed lemur, Vector-borne disease, Poland

## Abstract

**Background:**

*Anaplasma* are obligate intracellular bacteria and aetiological agents of tick-borne diseases of both veterinary and medical interest. The genus *Anaplasma* comprises six species: *Anaplasma marginale, Anaplasma centrale, Anaplasma ovis, Anaplasma phagocytophilum, Anaplasma bovis* and *Anaplasma platys.* They can infect humans, carnivores, ruminants, rodents, insectivores, birds and reptiles. The aim of this study was to present the first clinical case of granulocytic anaplasmosis in a captive ring-tailed lemur in Poland.

**Case presentation:**

A 4-year-old female lemur presented anorexia, epistaxis and tick infestation. The microscopic examination of a blood smear revealed morulae in neutrophils. Polymerase chain reaction test and sequencing of obtained PCR product confirmed infection by the GU183908 *Anaplasma phagocytophilum* strain. Therapeutic protocol included doxycycline (2.5 mg/kg p.o., b.i.d.) for 3 weeks and the lemur recovered within 24 h.

**Conclusions:**

This is the first report on granulocytic anaplasmosis in a ring-tailed lemur in Europe, indicating that *A. phagocytophilum* infection must also be considered in differential diagnosis in this animal species, especially in individuals with thrombocytopenia associated with *Ixodes ricinus* parasitism.

## Background

Tick-borne diseases (TBD) constitute a diversified group of diseases of increasing importance in human and veterinary medicine [1]. As showed by the observation of many authors, in Europe ticks are considered the most important of the arthropod zoonotic vectors [2–4], that are able to transmit such pathogens as *Anaplasma* spp., *Babesia/Theileria* spp. or *Borrelia* spp. *Anaplasma* spp. are pathogenic for several animal host species [5], while *Anaplasma phagocytophilum* is an emerging human pathogen in the USA and Europe [6, 7]. The clinical form of the disease is rarely reported in wild animals in captivity [8–10].

The aim of this study was to present the clinical case of granulocytic anaplasmosis in captive ring-tailed lemur (*Lemur catta)* in Poland.

## Case presentation

The observation took place in March 2020. The animal concerned was a female ring-tailed lemur *(Lemur catta)*, 4 years old, with a body weight of 4.2 kg, with signs of anorexia, weakness, epistaxis and uncoordinated gait. These clinical signs appeared four days before the animal was brought to the clinic. During a clinical examination two adult female *Ixodes ricinus* ticks were removed from the animal’s body. The ticks were identified on the basis of morphology using taxonomic keys [11]. The lemur came from a zoo in eastern Poland. The animal lived in a group of 6 lemurs. Two months before she had received fenbendazole (50 mg/kg p.o. for 3 days) as deworming treatment, but no ectoparasite prophylaxis had been applied. The animal was clinically examined and blood samples were collected for biochemical, haematological and molecular tests for tick-borne diseases (babesiosis/theileriosis/anaplasmosis/ehrlichiosis). The ticks found on the animal’s body were also tested for the above diseases using molecular methods.

DNA extractions from the blood samples and ticks for molecular tests were performed using a commercial DNA Genomic kit (A&A Biotechnology Gdańsk, Poland) following the manufacturer’s instructions. Subsequent PCR tests were performed according to the methods described by Skotarczak et al. [12], Altay et al. [13] and Adaszek and Winiarczyk [14] (Table 1). The final identification of tick-borne pathogens was performed by sequencing PCR products.

**Table 1:** PCR conditions and primers used in the PCR protocols for detecting *Anaplasma*/*Ehrlichia* spp., *Borrelia burgdorferi sensu lato* and *Babesia*/*Theileria* spp

Pathogen	Primers	Gene target	Amplicon size	PCR condition	Reference
*Anaplasma/Ehrlichia* spp.	EHR 521: (5′-TGT AGG CGG TTC GGT AAG TTA AAG-3′) EHR 747: (5′-GCA CTC ATC GTT TAC AGC GTG-3′)	16 S	247 bp	35 cycles: denaturation stage at 94 °C for 30 s, annealing at 56 °C for 30 s, elongation at 72 °C for 45 s	[14]
*Borrelia burgdorferi* s. l.	SC1: (5′-GCT GTC AGT GCG TCT TAA-3′) SC2: (5′-CTT AGC TGC TGC CTC CGT A-3′)	16 S	300 bp	35 cycles: denaturation stage at 94 °C for 60 s, annealing at 47 °C for 30 s, elongation at 72 °C for 90 s	[12]
*Babesia/Theileria* spp.	RLB R2: (5’-CTA AGA ATT TCA CCT CTG ACAGT-3’) RLB F2 (5’- GAC ACA GGG AGG TAG TGA CAAG-3’)	hypervariable V4 region of the 18 S rRNA	390–430 bp	40 cycles: denaturation stage at 94 °C for 35 s, annealing at 51 °C for 35 s, elongation at 72 °C for 35 s	[13]

**Fig. 1:**
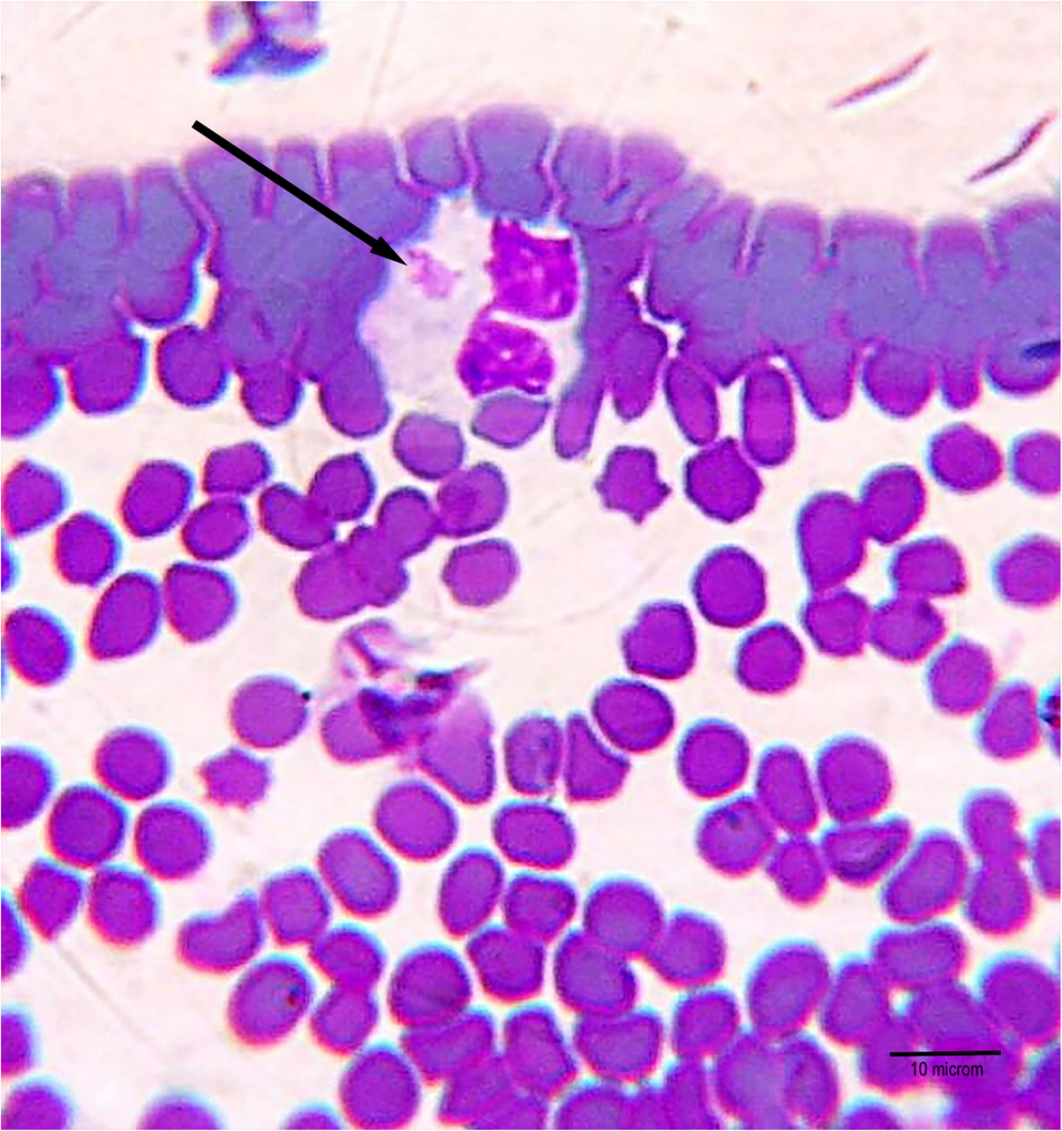
Presence of morulae of *A. phagocytophilum* inside neutrophil cell (marked with an arrow)

**Fig. 2:**
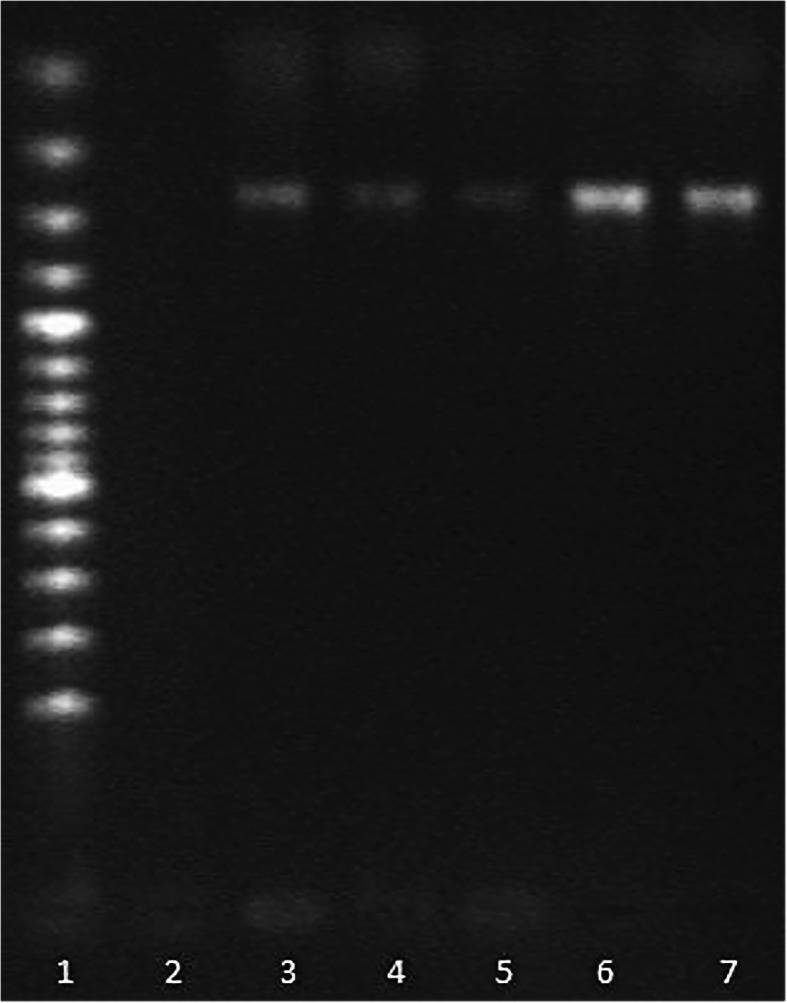
PCR amplification of a partial sequence of *A. phagocytophilum* 16 S RNA gene (product size 247 bp). Legend: lane 1 - molecular weight marker = 100 bp; lane 2 - negative control; lane 3 - positive control (*A. phagocytophilum* from human blood – National Reference Center for Borreliae of Max von Pettenkofer Institute of Ludwig Maximilian University Munich); lane 4 – amplification product of blood sample of lemur; lanes 5–7 - amplification products of ticks samples

The haematological and biochemical test results did not reveal any abnormalities, except for thrombocytopaenia (PLT = 61 × 10^9^/l; range 165–685 × 10^9^/l) [15]. The microscopic examination of the stained blood smear (Giemsa method) revealed morulae in the cytoplasm of circulating neutrophils suggestive of acute granulocytic anaplasmosis (Fig. 1). The PCR test revealed *Anaplasma* DNA in the lemur’s blood and in *I. ricinus* ticks collected from the lemur’s body (Fig. 2). The analysis of PCR product sequencing identified the Rickettsia species as *A. phagocytuphilum* GU183908 (100 % homology). Based on the microscopic blood smears and the molecular test results, the disease was caused by *Anaplasma phagocytophilum* infection. The treatment started with doxycycline (2.5 mg/kg p.o., b.i.d.) administered for three weeks. 24 h after the initiation of the treatment the lemur’s condition improved significantly: her appetite increased and normal gait was restored. Three days later the epistaxis had subsided. Two weeks following the initiation of the antibiotic treatment a sample of the animal’s blood was collected for a quick test to detect the presence of *A. phagocytophilum* antibodies (VetExpert, CaniV-4 Poland). The test result was positive. A control PCR test carried out after the next three weeks (according to the same procedure as previous) did not reveal genetic material of *A. phagocytophilum* in the animal’s blood. Also, no DNA of bacteria was found in the blood of the other five lemurs from the same institution. Six months after the beginning of therapy the antibodies for *A. phagocytophilum* were still be present in the animal’s blood.

## Discussion and conclusions

This article presents the first clinical case of granulocytic anaplasmosis in a lemur in Europe. *A. phagocytophilum* is one of the most prevalent tick-transmitted animal and human pathogen [7]. The main clinical disorders observed in the course of granulocytic anaplasmosis are fever, thrombocytopenia and lameness. Ticks and wildlife are the main reservoirs of these bacteria [8], but clinical disease in free-ranging as well as in captive wild animals appears to be rare. However wildlife may play a role in the transmission and maintenance of granulocytic anaplasmosis, either acting as a reservoir of the bacteria or amplifying host for human or domestic animals [16]. Therefore, it is important to identify the potential hosts and characterise the role in the epidemiology of various animal species in this disease in order to adequately evaluate the potential risks and to design proper strategies of control.

The main vector of the microorganisms in Europe is the tick *Ixodes ricinus* [17]. There are only a few specific reports regarding *A. phagocytophilum* infection in lemurs. Specific antibodies against *A. phagocytophilum* were found in the serum of lemurs from St. Catherine’s Island, Georgia, USA [18], whereas screening tests for infections, including *A. phagocytophilum*, conducted in the lemur population of Madagascar did not confirm a single case of the disease [19]. In Poland the disease was previously detected in horses [14], dogs [20] and cats [21], but never in exotic animals.

The definitive diagnosis of *A. phagocytophilum* infection in a ring-tailed lemur was confirmed by results of PCR and sequencing. The 16 S rRNA gene fragment of bacteria detected in the blood of the patient, as well as in the tick organism collected from the lemur’s body showed 100 % similarity with GU183908 uncultured *Anaplasma* species clone Lublin-1 from previous studies [14]. This suggests an endemic occurrence of this microorganism strain in Poland. The description of the presented case indicates that *A. phagocytophilum* infection must also be considered in differential diagnosis in exotic animals living in Poland, especially in individuals with thrombocytopenia associated with *Ixodes ricinus* parasitism.

## Data Availability

All data generated or analysed during this study are included in this published article.

## Abbreviations

TBD: Tick-borne diseases
kg: Kilogram
mg/kg: Milligrams per kilogram
*p.o.*: *Per os*
DNA: Deoxyribonucleic Acid
PCR: Polymerase Chain Reaction
*s.l.*: *Sensu lato*
*spp.*: *Species pluralis*
PLT: Platelets
b.i.d.: Bis in die
rRNA: Ribosomal Ribonucleic Acid
